# Impact of tumor burden on survival in patients with recurrent or metastatic head and neck cancer treated with immune checkpoint inhibitors

**DOI:** 10.1038/s41598-022-18611-z

**Published:** 2022-08-22

**Authors:** Takuma Matoba, Kiyoshi Minohara, Daisuke Kawakita, Gaku Takano, Keisuke Oguri, Akihiro Murashima, Kazuyuki Nakai, Sho Iwaki, Hiroshi Tsuge, Nobukazu Tanaka, Sae Imaizumi, Wataru Hojo, Ayano Matsumura, Koji Tsukamoto, Shinichi Esaki, Shinichi Iwasaki

**Affiliations:** 1 Department of Otorhinolaryngology, Head and Neck Surgery, Nagoya City University Graduate School of Medical Sciences, 1 Kawasumi, Mizuho-cho, Mizuho-ku, Nagoya, Aichi 467-8601 Japan; 2 Department of Otorhinolaryngology, Nagoya City University West Medical Center, Nagoya, Japan; 3 Department of Otorhinolaryngology, Konan Kosei Hospital, Konan, Japan; 4 Department of Head and Neck Medical Oncology, National Cancer Center Hospital East, Kashiwa, Japan; 5 Department of Head and Neck Surgery, The Cancer Institute Hospital of the Japanese Foundation for Cancer Research, Tokyo, Japan; 6 Department of Otorhinolaryngology, Anjo Kosei Hospital, Anjo, Japan; 7 Department of Otorhinolaryngology, Japanese Red Cross Aichi Medical Center Nagoya Daini Hospital, Nagoya, Japan

**Keywords:** Cancer, Oncology

## Abstract

Immune checkpoint inhibitors (ICIs) have become the standard treatment for recurrent or metastatic head and neck cancer (RM-HNC). However, many patients fail to benefit from the treatment. Previous studies have revealed that tumor burden predicts the efficacy of ICIs, but this association remains unclear for RM-HNC. We retrospectively analyzed 94 patients with RM-HNC treated with ICI monotherapy. We estimated the tumor burden using the baseline number of metastatic lesions (BNML) and the baseline sum of the longest diameters of the target lesions (BSLD), and evaluated the association between BNML, BSLD, and standardized uptake value (SUV) and clinical outcomes. The median progression-free survival (PFS) was 7.1 and 3.1 months in the low-BNML and high-BNML groups, respectively (p = 0.010). The median PFS was 9.1 and 3.5 months in the low-BSLD and high-BSLD groups, respectively (p = 0.004). Moreover, patients with high SUVmax levels had worse overall survival (OS) and PFS. BNML, BSLD, and SUVmax are useful prognostic factors in patients with RM-HNC treated with ICIs. Imaging examinations before ICI treatment are recommended to predict the efficacy of ICIs. If the tumor burden is high, cytotoxic anticancer agents may be administered concomitantly with or prior to ICI monotherapy.

## Introduction

The development of immune checkpoint inhibitors (ICIs) has changed the treatment strategies for several cancers. The results of the CheckMate 141 trial^1^ and Keynote 048 trial^2^ suggest that nivolumab and pembrolizumab—anti-programmed death-1 (PD-1) antibodies—are available for treatment of unresectable recurrent or metastatic head and neck cancer (RM-HNC). In real-world settings, these drugs have contributed to improved survival in patients with RM-HNC. However, > 50% of the patients fail to receive any clinical benefit from these drugs^3–5^.

Several predictive or prognostic factors have been evaluated^3–8^, but optimal timing for administering ICIs and optimal patient selection remain controversial. Some studies suggest that tumor burden, such as tumor size or number of metastatic lesions, is associated with progression-free survival (PFS) in patients with advanced cancers treated with ICIs^9–12^. Therefore, based on these studies, tumor burden may indicate the utility of ICIs or cytotoxic agents. Thus, to clarify the impact of tumor burden on survival in patients with RM-HNC, we retrospectively investigated patient charts of patients treated with ICIs at our institute. Specifically, we evaluated the association between the baseline number of metastatic lesions (BNML), the baseline sum of the longest diameters of the target lesions (BSLD), and maximum standardized uptake value (SUVmax) and clinical outcomes such as survival and response to anti-PD-1 monotherapy.

## Results

### Patient characteristics and clinical outcomes of total population

Ninety-four patients treated with anti-PD-1 monotherapy were enrolled in this study. The median age of patients was 70 (37–90) years, and a majority were men (76 patients, 80.9%). Of these 94 patients, 65 and 29 were treated with nivolumab and pembrolizumab as the first ICI treatment, respectively. ICI was the first systemic therapy, 1st line, for RM-HNC in 64 patients, the 2nd line in 21 patients and the 3rd line or more in 9 patients. The best overall response (BOR) was complete response (CR) in one patient, partial response (PR) in 18 patients, stable disease (SD) in 42 patients, and progressive disease (PD) in 33 patients (Table 1).

**Table 1:** Patient characteristics.

	Total (N = 94)	
	N	%
**Age**		
≤ 70 (median)	48	51.1
> 70 (median)	46	48.9
**Sex**		
Male	76	80.9
Female	18	19.1
**ECOG PS**		
0, 1	70	74.5
2, 3	24	25.5
**Regimen**		
Nivolumab	65	69.1
Pembrolizumab	29	30.9
**ICI treatment line**^**a**^		
1st	64	68.1
2nd	21	22.3
3rd or more	9	9.6
**BOR**		
Non-PD	61	64.9
(CR)	(1)	(1.1)
(PR)	(18)	(19.2)
(SD)	(42)	(44.7)
PD	33	35.1
**BNML**		
One	42	44.7
More	52	55.3
**BSLD**		
≤ 28	33	35.1
> 28	61	64.9
**SUVmax**		
≤ 12.93	23	24.5
> 12.93	25	26.6
unknown	46	48.9

*ECOG* Eastern Cooperative Oncology Group, *PS* performance status, *ICI* immune checkpoint inhibitor, *BOR* best overall response, *PD* progressive disease, *BNML* baseline number of metastatic lesions, *BSLD* baseline sum of target lesions' longest diameters, *SUV* standardized uptake value.

^a^ICI treatment line was counted as the number of systemic chemotherapy for recurrent or metastatic head and neck cancer.

The median overall survival (OS) and PFS of all patients were 14.0 months (95% confidence interval [CI]: 11.9–18.3) and 4.9 months (95% CI 3.2–6.9), respectively. One-year OS of all patients was 60.7% (95% CI 48.7–70.7%) and the estimated 6-months PFS was 43.5% (95% CI 33.1–53.4%). The overall response rate was 20.2% (95% CI 12.6–29.8%) and disease control rate was 64.9% (95% CI 54.4–74.5%).

### Tumor burden and clinical outcomes

BNML was 1 in 42 cases, 2 in 31 cases, and ≥ 3 in 21 cases. The median BSLD was 43 (0–426) mm. By performing receiver operating characteristic (ROC) curve analysis, the cutoff value of BNML for predicting survival and classifying patients into low-BNML (BNML = 1; 42 patients [44.7%]) and high-BNML (BNML ≥ 2; 52 patients [55.3%]) groups was 1. The cutoff value of BSLD was 28 mm, determined by performing ROC curve analysis, and patients were classified into low-BSLD (BSLD ≤ 28; 33 patients [35.1%]) and high-BSLD (BSLD > 28; 61 patients [64.9%]) groups.

**Figure 1:**
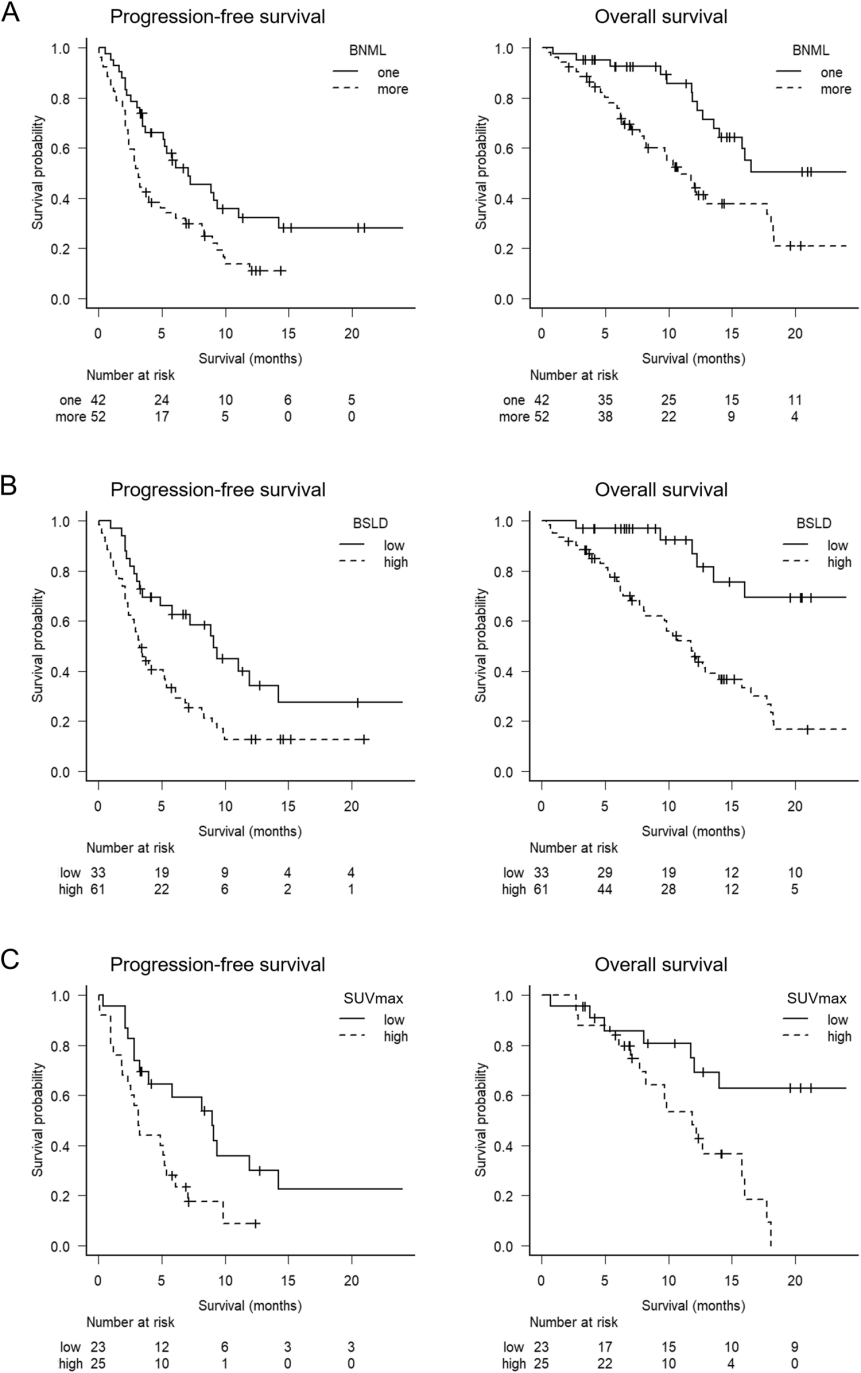
Progression-free and overall survival stratified by the baseline number of metastatic lesions (**A**), the baseline sum of the longest diameters of the target lesions (**B**), and maximum standardized uptake value (**C**). (**A**) Kaplan–Meier curves of Progression-free survival (PFS) and Overall survival (OS) stratified by the baseline number of metastatic lesions (BNML). Patients with BNML was one (N = 42) had significantly better PFS than those with BNML was more than one (N = 52) (6-months PFS: 55.0% [95% CI 38.2–69.0%] vs. 34.0% [95% CI 21.5–47.0%], p = 0.008). Patients with BNML was one (N = 42) had significantly better OS than those with BNML was more than one (N = 52) (1-year OS: 78.6% [95% CI 59.8–89.4%] vs. 47.0% [95% CI 31.5–60.9%], p = 0.002). (**B**) Kaplan–Meier curves of PFS and OS stratified by baseline sum of the longest diameters of the target lesions (BSLD). Patients with BSLD ≤ 28 mm (low, N = 33) had significantly better PFS than those with BSLD > 28 (high, N = 61) (6-months PFS: 62.6% [95% CI 43.5–76.8%] vs. 33.2% [95% CI 21.6–45.3%], p = 0.003). Patients with BSLD ≤ 28 mm (low, N = 33) had significantly better OS than those with BSLD > 28 (high, N = 61) (1-year OS: 86.9% [95% CI 63.7–95.7%] vs. 47.8% [95% CI 33.8–60.6%], p < 0.001). (**C**) Kaplan–Meier curves of PFS and OS stratified by maximum standardized uptake value (SUVmax) of PET. Patients with SUVmax ≤ 12.93 (low, N = 23) had significantly better PFS than those with SUVmax > 12.93 (high, N = 25) (6-months PFS: 59.2% [95% CI 35.9–76.5%] vs. 28.0% [95% CI 12.4–46.0%], p = 0.014). Patients with SUVmax ≤ 12.93 (low, N = 23) had significantly better OS than those with SUVmax > 12.93 (high, N = 25) (1-year OS: 75.0% [95% CI 49.7–88.9%] vs. 48.1% [95% CI 25.6–67.5%], p = 0.003).

The median PFS was 7.1 months (95% CI 3.7–11.1) and 3.1 months (95% CI 2.3–4.9) in the low-BNML and high-BNML groups, respectively (p = 0.008). The median OS was 31.5 months (95% CI 13.5–NA) and 10.7 months (95% CI 7.7–17.7) in the low-BNML and high-BNML groups, respectively (p = 0.002) (Fig. 1A). The median PFS was 9.1 months (95% CI 4.9–14.2) and 3.5 months (95% CI 2.4–5.2) in the low-BSLD and high-BSLD groups, respectively (p = 0.003). The median OS was 31.5 months (95% CI 16.0–NA) and 11.8 months (95% CI 8.0–14.0) in the low-BSLD and high-BSLD groups, respectively (p < 0.001) (Fig. 1B).

In univariate analysis, patients with worse BOR (hazard ratio [HR]: 15.20, 95% CI 7.49–30.84, p < 0.001), high-BNML (HR: 1.91, 95% CI 1.17–3.12, p = 0.010), or high BSLD (HR: 2.17, 95% CI 1.27–3.69, p = 0.004) had worse PFS (Table 2). Additionally, patients with worse Eastern Cooperative Oncology Group (ECOG) performance status (PS) (HR: 3.24, 95% CI 1.70–6.17, p < 0.001), worse BOR (HR: 3.51, 95% CI 1.95–6.32, p < 0.001), high-BNML (HR: 2.58, 95% CI 1.39–4.80, p = 0.003), or high-BSLD (HR: 4.47, 95% CI 1.99–10.03, p < 0.001) had worse OS (Table 2). Multivariate analysis revealed that patients with high-BNML (HR: 1.98, 95% CI 1.19–3.29, p = 0.008) or high-BSLD (HR: 2.57, 95% CI 1.44–4.58, p = 0.001) had worse PFS (Table 2). Furthermore, patients with worse ECOG PS (HR: 2.80, 95% CI 1.45–5.37, p = 0.002), high-BNML (HR: 2.30, 95% CI 1.21–4.36, p = 0.011), or high-BSLD (HR: 4.62, 95% CI 1.99–10.71, p < 0.001) had worse OS after adjustment for potential confounders (Table 2).

**Table 2:** The association between the tumor burden and clinical outcomes in patients with recurrent or metastatic head and neck cancer treated with immune checkpoint inhibitors.

	N	Univariate analysis						Multivariate analysis					
		Progression-free survival			Overall survival			Progression-free survival			Overall survival		
		HR	95% CI	p-value	HR	95% CI	p-value	HR	95% CI	p-value	HR	95% CI	p-value
**BNML**													
One	42	1.00	–	–	1.00	–	–	1.00	–	–	1.00	–	–
More	52	1.91	1.17–3.12	0.010	2.58	1.38–4.80	0.002	1.98	1.19–3.29	0.008	2.19	1.15–4.18	0.017
**BSLD**													
≤ 28	33	1.00	–	–	1.00	–	–	1.00	–	–	1.00	–	–
> 28	61	2.17	1.27–3.69	0.004	4.47	1.99–10.03	< 0.001	2.57	1.44–4.58	0.001	3.10	1.30–7.41	0.011
**SUVmax**													
≤ 12.93	23	1.00	–	–	1.00	–	–	1.00	–	–	1.00	–	–
> 12.93	25	2.34	1.16–4.70	0.017	3.65	1.46–9.16	0.006	3.85	1.71–8.65	0.001	1.93	0.60–6.25	0.271

Adjusted by age, sex, Eastern Cooperative Oncology Group performance status. In analysis for overall survival, adjusted also by best overall response for anti-PD-1 monotherapy.

*HR* hazard ratio, *CI* confidence interval, *BNML* the baseline number of metastatic lesions, *BSLD* the baseline sum of the longest diameters of the target lesions, *SUV* standardized uptake value.

We also evaluated the association between tumor burden and response to anti-PD-1 monotherapy. Univariate logistic regression analyses identified high-BNML (odds ratio [OR]: 2.54, 95% CI 1.04–6.22, p = 0.042) and high-BSLD (OR: 3.57, 95% CI 1.29–9.90, p = 0.014) as risk factors for worse disease control rate. After adjusting for potential confounders, high-BNML (OR: 2.81, 95% CI 1.10–7.20, p = 0.031) and high-BSLD (OR: 4.71, 95% CI 1.53–14.50, p = 0.007) were validated as risk factors for worse disease control rate (Table 3).

**Table 3:** The association between the tumor burden and the response to anti-PD-1 monotherapy in patients with recurrent or metastatic head and neck cancer.

	N	Univariate analysis			Multivariate analysis		
		Disease control rate			Disease control rate		
		HR	95% CI	p-value	HR	95% CI	p-value
**BNML**							
one	42	1.00	–	–	1.00	–	–
more	52	2.54	1.04–6.22	0.042	2.81	1.10–7.20	0.031
**BSLD**							
≤ 28	33	1.00	–	–	1.00	–	–
> 28	61	3.57	1.29–9.90	0.014	4.71	1.53–14.50	0.006
**SUVmax**							
≤ 12.93	23	1.00	–	–	1.00	–	–
> 12.93	25	4.28	1.16–16.60	0.030	18.60	1.95–178.00	0.011

Adjusted by age, sex, Eastern Cooperative Oncology Group performance status.

*HR* hazard ratio, *CI* confidence interval, *BNML* the baseline number of metastatic lesions, *BSLD* the baseline sum of the longest diameters of the target lesions, *SUV* standardized uptake value.

### Maximum standardized uptake value (SUVmax) and clinical outcomes

Of the 94 patients, 48 underwent positron emission tomography (PET) between the appearance of recurrent or metastatic lesions and initial administration of nivolumab or pembrolizumab. Median SUVmax of these 48 patients was 13.07 (range: 3.50–31.70). The cutoff value of SUVmax determined by performing ROC curve analysis was 12.93. When we compared survival outcomes between patients with high SUVmax (> 12.93; 25 patients) and those with low SUVmax (≤ 12.93; 23 patients), patients with high SUVmax had worse PFS and OS. The median PFS was 9.0 months (95% CI 3.2–14.2) and 3.1 months (95% CI 1.87–5.40) for patients with low SUVmax and high SUVmax, respectively (p = 0.014). The median OS was 38.1 months (95% CI 11.8–NA) and 11.9 months (95% CI 7.7–16.0) for patients with low SUVmax and high SUVmax, respectively (p = 0.003) (Fig. 1C). In univariate analysis, high SUVmax was associated with worse PFS (HR: 2.34, 95% CI 1.16–4.70, p = 0.017) and OS (HR: 3.65, 95% CI 1.46–9.16, p = 0.006) (Table 2). In the multivariate analysis, patients with high SUVmax had worse PFS (HR: 3.85, [95% CI 1.72–8.65], p = 0.001) (Table 2). We also evaluated the association between SUVmax and response to anti-PD-1 monotherapy. In univariate (OR: 4.28, 95% CI 1.16–16.60, p = 0.030) and multivariate (OR: 18.60, 95% CI 1.95–178.00, p = 0.011) logistic regression analyses, high SUV_max_ was found to be a risk factor for worse disease control rate (Table 3).

## Discussion

In the present study, we evaluated the impact of BNML, BSLD, and SUVmax on clinical outcomes in patients with RM-HNC treated with ICIs; the analyses identified high BNML, high BSLD, and high SUVmax as risk factors for worse survival rates and worse disease control rates.

To the best of our knowledge, this is the first study to clarify that high BNML, high BSLD, and high SUVmax are associated with worse clinical outcomes in patients with RM-HNC treated with nivolumab or pembrolizumab. A study suggested that pretreatment tumor size may affect response to nivolumab in patients with head and neck squamous cell carcinoma^10^; but, we analyzed about both tumor size and number of lesions, as well as both nivolumab and pembrolizumab, to evaluate the impact of tumor burden on the clinical outcomes of ICIs.

The impact of tumor burden on survival has been reported for several cancers^9,11,12^. Miyawaki et al. reported that tumor burden can predict the efficacy of PD-1/PD-L1 inhibitor monotherapy against non-small cell lung cancer^9^. In this study, the values of BNML and BSLD were commonly available in clinical settings and were strongly associated with the clinical outcomes of PD-1/PD-L1 inhibitor monotherapy. Therefore, we adopted these parameters to evaluate tumor burden.

The mechanism by which a high tumor burden diminishes ICI efficacy remains unclear. Huang et al. demonstrated that a lower proportion of reinvigorated CD8^+^ T cells and tumor burden was associated with worse clinical outcomes in patients with melanoma treated with anti-PD-1 therapy^13^. Immune phenotypes of immune-inflamed, immune-excluded, and immune-deserted tumors have been described to be correlated with response to immunotherapy^14^. Therefore, the infiltration of immune cells may be regulated by a large tumor volume. However, tumor volume failed to correlate with immune phenotypes in one study^15^; thus, further investigation is needed.

We also evaluated the impact of SUVmax on clinical outcomes. In other cancers, SUVmax has been reported as a prognostic factor or predictor in patients treated with ICIs^16,17^. Ichiki et al. demonstrated that aggressive cancers, such as those with high SUVmax, may not be suitable for ICIs^16^. Approximately 50% of patients in the present study were not included in the SUVmax analysis, but the results indicate an association between SUVmax and clinical outcomes in patients with RM-HNC treated with ICIs.

This study has several strengths. First, the number of patients who received anti-PD-1 monotherapy and were included in the study (n = 94) was relatively large. We found statistically significant differences in clinical outcomes between the high and low tumor burden groups. Second, we used both BNML and BSLD to evaluate the tumor burden. The BSLD is a useful marker, but unmeasurable lesions could not be evaluated. Therefore, we used both BNML and BSLD to minimize the potential variation from the actual tumor burden. Third, we evaluated the impact of SUVmax. The analysis suggests the potential use of SUVmax as a marker for predicting ICI efficacy. As a high SUVmax value may indicate tumor aggressiveness, SUVmax is a potential tool for evaluating tumor burden.

This study has some limitations. First, it was a retrospective study conducted at a single institution. Second, we could not evaluate all metastatic lesions. The unmeasurable lesions, such as those with unclear borders or small lesions, were excluded from analyses. Third, the follow-up duration was too modest to evaluate long-term survival outcomes. Forth, although we analyzed both survival outcomes and the best overall response, it is difficult to strictly define the tumor burden as a predictive factor or prognostic factor for ICI treatment, because this is not a comparison study between ICIs and other treatment^18^. Therefore, a prospective, multicenter study is warranted to evaluate the impact of clinical tumor burden on survival in patients with RM-HNC treated with ICIs.

In conclusion, the study suggests that BNML, BSLD, and SUVmax may be prognostic factors in patients with RM-HNC treated with nivolumab or pembrolizumab monotherapy. We recommend to perform imaging examinations, including computed tomography (CT), magnetic resonance imaging (MRI), and PET before administration of ICIs to assess tumor spread and predict efficacy of ICIs. The study also indicates that a high tumor burden may qualify for chemotherapy with cytotoxic agents to be administered with or prior to ICI monotherapy.

## Methods

### Patients

We retrospectively analyzed 94 patients with RM-HNC who received nivolumab or pembrolizumab monotherapy at the Nagoya City University Hospital between July 2017 and September 2021.

We defined measurable tumor lesions as those with the longest diameter > 10 mm on CT or MRI. Measurable neck lymph nodes were defined as those with maximum minor axis diameter > 10 mm. We estimated the tumor burden using BNML and BSLD^9^. We defined BNML as both measurable and unmeasurable lesions, and multiple metastases in the same region were counted as single lesions.

We also measured SUVmax using PET and assessed its impact on survival. Patients who did not undergo PET in the duration between the appearance of recurrent/metastatic lesions and initial administration of nivolumab or pembrolizumab were excluded from analyses of SUVmax.

### Treatment and follow-up

Between July 2017 and January 2020, nivolumab was administered at a dosage of 3 mg/kg every two weeks. Between February 2020 and April 2021, the dosage was 240 mg/body every two weeks. In stable cases, nivolumab was administered at a dosage of 480 mg/body every four weeks since September 2020. Pembrolizumab was administered at a dosage of 200 mg/body every three weeks in standard cases and 400 mg/body every six weeks in stable cases. The response to these therapies was evaluated according to the Response Evaluation Criteria in Solid Tumor (RECIST) version 1.1^19^, using CT or MRI, every 8–12 weeks. Patients with clinically obvious disease progression were diagnosed with PD even when images were not evaluated. Chemotherapy with cytotoxic agents was administered to patients diagnosed with PD. Follow-up was continued until death or the cutoff date (November 30, 2021).

### PET image acquisition

The image acquisition methods were same as previous studies^20,21^. Patients received fasting at least 4 h and then administered a standardized dose of 3.5 MBq 18-fluorodeoxyglucose (FDG) per kilogram body weight. After FDG injection, patients were kept in a lying position for 60 min prior to image acquisition. FDG-uptake parameters were evaluated using Advantage Workstation 4.6 software program the PET VCAR (GE Healthcare, Chalfont, UK). SUVmax was calculated automatically using a standard formula [maximum activity in region of interest ÷ (injected dose × body weight)].

### Statistical analysis

OS was calculated from the start of anti-PD-1 antibody monotherapy until death from any cause. PFS was calculated from the start of anti-PD-1 antibody monotherapy until disease progression or death from any cause. The median OS and PFS were evaluated using the Kaplan–Meier method and log-rank test. The impact of BNML, BSLD, and SUVmax on survival was assessed using univariate and multivariate analyses with Cox proportional hazards models. The cutoff value for each factor was determined by performing ROC curve analyses. The correlations between BNML, BSLD, and response to anti-PD-1 monotherapy were assessed using univariate and multivariate logistic regression analyses. A p < 0.05 was considered to be statistically significant. Age, sex, ECOG PS, and BOR for anti-PD-1 antibody monotherapy were defined as potential confounders in multivariate analysis of OS. Whereas, age, sex, and ECOG PS were defined as confounders in multivariate analysis of PFS.

All analyses were performed using EZR (Saitama Medical Center, Jichi Medical University, Saitama, Japan), a graphical user interface for R (The R Foundation for Statistical Computing, Vienna, Austria, version 3.5.0). EZR, a modified version of the R commander (version 2.7–1), was designed to incorporate statistical functions frequently used in biostatistics.

### Ethical approval

This study was approved by the Institutional Review Board of the Nagoya City University Graduate School of Medical Sciences (Accession No. 60-21-0001). As this was a retrospective, non-intervention study, patients could reject participation by opting out to an announcement on the Nagoya City University Hospital’s website, and the requirement of written informed consent was waived. This study was conducted in accordance with the principles of the Declaration of Helsinki.

## Data Availability

The datasets generated and/or analyzed during the study are available from the corresponding author upon reasonable request.
